# Optimization of the Convolutional Neural Networks for Automatic Detection of Skin Cancer

**DOI:** 10.1515/med-2020-0006

**Published:** 2020-01-13

**Authors:** Long Zhang, Hong Jie Gao, Jianhua Zhang, Benjamin Badami

**Affiliations:** 1 Department of medical equipment, People’s hospital of Zhengzhou University, Zhengzhou, 450001, China; 2 Institute of Medical Engineering Technology and Data Mining, Zhengzhou University, Zhengzhou, 450001, China; 3 University of Georgia, Athens, USA

**Keywords:** Computer-aided diagnosis, Skin cancer detection, Convolutional neural networks, Whale optimization algorithm, Image segmentation

## Abstract

Convolutional neural networks (CNNs) are a branch of deep learning which have been turned into one of the popular methods in different applications, especially medical imaging. One of the significant applications in this category is to help specialists make an early detection of skin cancer in dermoscopy and can reduce mortality rate. However, there are a lot of reasons that affect system diagnosis accuracy. In recent years, the utilization of computer-aided technology for this purpose has been turned into an interesting category for scientists. In this research, a meta-heuristic optimized CNN classifier is applied for pre-trained network models for visual datasets with the purpose of classifying skin cancer images. However there are different methods about optimizing the learning step of neural networks, and there are few studies about the deep learning based neural networks and their applications. In the present work, a new approach based on whale optimization algorithm is utilized for optimizing the weight and biases in the CNN models. The new method is then compared with 10 popular classifiers on two skin cancer datasets including DermIS Digital Database Dermquest Database. Experimental results show that the use of this optimized method performs with better accuracy than other classification methods.

## 1 Introduction

Skin cancer involves abnormal changes in the outer layer of the skin. This cancer is the most prevalent cancer in the world and contains about 75% of the world’s cancer. Although most people with skin cancer are healed, it is still a major concern due to its high prevalence [1]. Most skin cancers grow only locally and invade adjacent tissues, but some of them, particularly melanoma (cancer of the pigment cells), which is the rarest type of skin cancer, may spread through the circulatory system or lymphatic system and reach the farthest points of the body [2]. Melanoma forms the highest percentage of probability among different types of skin cancer [3, 4, 5, 6, 7]. On average, 4740 males and 2490 females died in 2019 due to melanoma [8]. Melanoma is more prevalent in some areas, especially in western regions and countries. According to the findings, the diagnosis of melanoma in the initial stages can significantly reduce the mortality due to this cancer; but since the diagnosis of this disease at an early stage, even by specialists, is difficult, it will be very helpful to provide a method to early diagnosis of the melanoma or skin cancer [9, 10, 11, 12].

In recent years, with the advancement of technology, particularly artificial intelligence, suitable methods have been developed for this issue. In the meantime, image processing techniques are progressing as successful methods [9, 13, 14, 15, 16]. The application of image processing and computer vision for automatically identifying the patterns like cancer from images reduces human errors and increases the speed of detection. In addition, the importance of medical image processing can be considered as it helps physicians and radiologists to more easily diagnose the disease, thus protecting the patient against irreparable risks that will co me about. Artificial Neural Networks (ANNs) are one of the popular methods used in image processing. ANN is inspired by the intricate structure of the human brain, in which millions of neurons (cells) communicate with each other (synapse) to solve problems or store information. These networks are a collection of different models that are proposed by mathematicians and engineers to simulate a part of brain function [15, 16, 17, 18, 19, 20, 21].

The system is made up of a large number of extravagant processing elements called neurons that work together to solve a problem. Learning in natural systems occurs adaptively; this means that there is a change in the synapse as a result of learning. Recently, important developments are proposed based on new kinds of neural networks for analyzing visual systems. CNN is a trail of deep neural networks which is usually used on image or speech analyzes in machine learning [22, 23, 24].

In addition to different applications of CNN’s in image processing, they have especially promising performance in different medical image problems like lesion classification [25], breast cancer [26], tumor diagnosis [26], brain analysis [27], panoptic analysis [28], and MR images fusion [29]. In the mentioned examples of CNN applications, the image has to be first divided into a lot of small superpixels and then the methods have to be performed on all of the superpixels. From the literature, it is observed that using CNN models improves the diagnosis system performance [30]. A part of the training step in the neural networks is to find the optimal solution to fit the target problem based on internal weights which is usually established by the back propagation (BP) algorithm. BP is a classic method that evaluates the error on each training pairs and adjusts the neurons weights to fit the desired output [31]. The error minimization is established by the gradient descent algorithm as it minimizes the cross-entropy loss in the image. This is a complicated optimization problem which needs high cost for solving it.

Recently, the utilization of meta-heuristics in different applications is extensively increasing. One of these applications can be to use them for the cross-entropy loss minimization [32]. In the recent years, several kinds of meta-heuristic algorithms have been introduced. In 2016, Mirjalili and Lewis proposed a new meta-heuristic method called whale optimization algorithm [33]. The whale optimization algorithm is an inspiration of the bubble net hunting strategy of the humpback whales. Despite being new, it has good results for different applications [34, 35, 36, 37, 38]. Here, this algorithm is employed for the cross-entropy loss minimization of skin cancer images to improve the method efficiency. In the present study, the whale optimization algorithm is employed for the diagnosis of cancer images. The main purpose here is to optimize the weights of any layer of CNN. The proposed optimization algorithm shows suitable improvements about optimal training of the CNN.

The main structure of the paper is given in the following. Section “Materials and Methods” describes comprehensive explanations about materials and methods including convolutional neural networks (CNN) and whale optimization algorithm (WOA).

In Section “The Proposed WOA based CNN”, the new optimized convolutional neural network based on a whale optimization algorithm is presented. Section “Dataset Description” briefly presents the dataset which is considered for performance analysis.

Section “Implementation Results” investigates the experimental results by performing a comparison between the proposed method and 10 popular cancer detectors, and the conclusions of the paper are given in section “Conclusions”.

## 2 Materials and Methods

In the following, a general description about the CNN and how to optimize them will be described.

### 2.1 Convolutional neural networks

Here, the membranous neurons respond to the motive in bounded areas called *receptive fields*. The receptive field for each neuron partially overlaps until the visual field is tiled. The reply to the every single neuron to the motive can be mathematically approximated by a convolution operation [39,40].

Convolutional neuron layers contain the most principal part of a CNN. For classification applications like image classification, multiple 2D matrices can be considered as the input and the output of the convolutional layer. It is important to note that there is no restriction about the equality in the number of the input and the output matrices.

In this step, local feature extraction has been applied to extract the regional characteristics of the original image. The main purpose of the learning procedure is to obtain some kernel matrices to get better prominent features to be utilized in image classification. The BP algorithm can be used here for optimizing the network connection weights. The convolution in this layer is performed by a sliding window. Afterward, a vector has been generated based on the sliding window and the dot product and the weights are added up.

Then, an activation function is utilized for each neuron which is often a rectified linear unit (ReLU), with a function *f*(*x*) = max(*x*, 0) [41]. This process has been implemented on the original image. For more scale reduction of the output, another process called max pooling has been employed; here, only the highest value is reported to the subsequent layer of the sliding grid. After initializing the structure of a CNN, an optimization method will be required to fit the target problem based on internal weights. This process is usually applied by the BP algorithm. In BP, the err or on each training pairs is evaluated and then it is employed to adjust the weights of the neurons to fit the desired output [6, 31]. BP uses a gradient descent algorithm for the error minimization. The gradient descent is a method based on minimizing the cross-entropy loss as the fitness function [42]. The proposed fitness function is given in the following.

(1) $$L=\sum_{j=1}^{N}\sum_{i=1}^{M}-d_{j}^{\left(i\right)}\log z_{j}^{\left(i\right)}$$

where, $d_{j}=\left(0,\ldots ,0,\underset{k}{\underset{︸}{1,\ldots ,1}},0,\ldots ,0\right)$describes the desired output vector and Z_j_is the obtained output vector of the *m^th^* class.

The softmax function is illustrated in the following formula:

(2) $$Z_{j}^{\left(i\right)}=\frac{e^{f_{j}}}{\sum_{i=1}^{M}e^{f_{i}}}$$

where, *N* is the number of samples.

The function *L* can be modified by the weight penalty to include a γvalue, to keep the values of the weights from getting larger:

(3) $$L=\sum_{j=1}^{N}\sum_{i=1}^{N}-d_{j}^{\left(i\right)}\log z_{j}^{\left(i\right)}+\frac{1}{2}\gamma \sum_{K}\sum_{L}W_{k,l}^{2}$$

where, *W_k_* describes the connection weight, *k* in layer la and *L* and *K* are the total number of layers and the layer *l* connections, respectively.

Although CNN has been introduced as a powerful classification tool, designing an optimal structure for its layout is a significant problem: most of the designed layouts are based on trials and errors.

**Figure 1:**
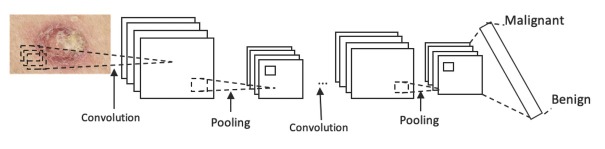
A simple skin cancer detection using ordinary CNN

Recently, there are some new works which have introduced modifications based on meta-heuristic algorithms [43, 44]. Fig. 1 shows a simple skin cancer detection using ordinary CNN.

In the fig. 1 the convolution layer evaluates the output of the neurons that are connected to the local area at the input. The calculation is performed by the point multiplication between the weights of each neuron and the area they are connected to (the activation mass). The main purpose of the pooling layer is also to subsample the input image to reduce the computational load, memory, and the number of parameters (over fitting). Reducing the size of the input image also causes the neural network to be less sensitive to image displacement (independent of the position).

The WOA is a new stochastic optimization method which is derived from the hunting process of whales [33]. Like any evolutionary method, WOA starts with a random population set (candidate solutions) to search and find the global optimum (maximum or minimum) solution for the problem. The algorithm continues to improve and update the solution based on its structure until the optimum value is satisfied.

The basic difference between the WOA and other meta-heuristic methods is how the WOA rules develop and update the solution.

**Figure 2:**
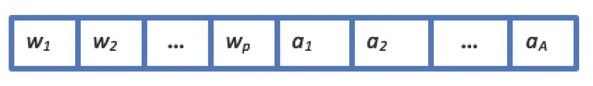
The search agent vector assigning of WOA on the CNN

The WOA is inspired from the whale’s trap and attack hunting strategy; the use of bubbles in spiral movement around the prey with which the trap is formed is given the name “*bubble-net feeding behavior*”. The behavior of the bubble-net feeding process is shown in Fig. 2. From the

figure 2, it is clear that the humpback whale first creates bubbles around the prey. This process is performed by spiral motion of the whale. Afterward, it attacks the prey. This process comprises the main contributor of the WOA. The explained created bubble-net system is mathematically defined as follows:

(4) $$X\left(t+1\right)=\{{}_{{}_{D^{'}e^{bl}\cos\left(2\pi t\right)+X^{*}\left(t\right)\;\;\;\;\;\;\;\;\;\;\;\;\;\;\;\;p\geq \;0.5}}^{\;\;\;\;\;\;\;\;X^{*}\left(t\right)-AD\;\;\;\;\;\;\;\;\;\;\;\;\;\;\;\;p<0.5}$$

(5) $$D^{'}=\left|CX^{*}\left(t\right)-X\left(t\right)\right|$$

(6) A = 2ar−a

(7) C=2r 

where, *p* and *r* are random constants and are bounded between [0, 1], *l* is a random constant in the interval [-1, 1], *t* illustrates the present iteration, D'describes the distance for the *i^th^* whale from the prey (the best solution), *b* defines the logarithmic shape of the spiral motion, *a* is a linear descent from 2 to 0 over the iteration.

In the above equation, the first term models the encircling process and the second term models the bubble-net process. In WOA, these two terms comprise the exploitation and exploration terms of the algorithm [33].

As it is mentioned before, the WOA starts with a random population. The solutions are then updated in each iteration in order for mathematical modeling of the bubble net hunting and the prey encircling. Here, to ensure the convergence of the algorithm, the best solution improves the position of the agents when |X>1. Otherwise, the best solution will play the rule of the pivot point. In the following, the general pseudo code for the WOA is given.

***Start***

Initializing the whale population X_i_

Initializing A, C, and a

Evaluate the cost value of each agent

X^*^ shows the best solution in the current iteration

***Apply WOA*****:

t=1

***while*** *t* ≤*max iteration*:

***for*** *all the agents*

***if*** *|A|* ≤ *1 **then***

Update the position of the agents

***else if*** *|A|* ≥ *1 **then***

Find a random search agent X_rand_

Update the position of the agents

***end if***

***end for***

Update A, C, and a

Update X*

t = t + 1

***end while***

return X*

***End***

### 2.2 The Proposed WOA based CNN

In the present study, a different strategy is utilized specifying the number of hyper-parameters; not only the most appropriate hyper-parameters for the CNN at the moment can be considered, it can also take into account the time to run each moment.

As mentioned before, the primary purpose of this research is to design an optimization based technique for skin cancer classification. The main idea here is to utilize an optimized method to improve the system accuracy. Candidate solutions in the proposed optimized classification problem are a sequence of integers. In this method, at first, the minimum (min) and the maximum (max) limitations for the algorithm is determined to prevent the system errors. In this problem, max describes the size of the sliding window and min is 2. Here, the constant 2, presents the minimum value that is acceptable for the max pooling where no lower size exists.

The other point which should be considered is that the value of the input data should be greater than the sliding window. Afterward, a group of solutions is randomly generated. In this problem, the initial population is set to 150, where the hyper-parameters settings of the CNN are described by the individuals, occurring within 10 integer values. The search agent vector for the proposed CNN is shown in Fig. 2.

Afterward, the solutions are evaluated. Here, the halfvalue precision for the proposed optimized CNN is considered as the cost function on a skin cancer validation process. It is important to know that the general strategy has high computational cost; each member of the population describing the CNN requires training on the skin cancer dataset with applying back propagation algorithm for 1500 iterations.

After initial population generating and evaluation of the initial cost, the position of the search agents are updated based on the parameters like prey encircling and bubble net hunting and the process repeats until the stop criteria are achieved.

The designed optimized system was tested on the DermIS and the Dermquest databases based on the minimization of the MSE value for validating and testing. In the following, more explanations are described. Weights and biases are two important parameters which are used for optimizing the structure of the CNN. Therefore, in this research, these two features have been selected for optimizing, such that:

(8) $$W\;=\left\{w_{1},w_{2},\ldots ,w_{p}\right\}$$

(9) $$A=\left\{a_{1},a_{2},\ldots ,a_{A}\right\}$$

(10) $$w_{n}=\left\{w_{1n},w_{2n},\ldots ,w_{Ln}\right\}$$

(11) $$\begin{array}{l} b_{n}=\left\{b_{1n},b_{2n},\ldots ,b_{Ln}\right\}\\ \;\;\;\;\;\;\;\;\;\;l=1,2,\ldots ,L\\ \;\;\;\;\;\;\;\;\;n=1,2,\ldots ,A \end{array}$$

where, *A* and *L* are the total number of agents and the total number of layers respectively, *l* describes the layer index, *n* describes the number of the agent, and *w_in_* describes the value of the weight in layer *i*. In other words, the total parameters for optimizing can be described by the following vector:

(12) $$W_{n}=\left\{W,A\right\}$$

Fig.2. shows these assignments.

A simplified measured error between the reference and the system output is given below.

(13) $$E=\frac{1}{T}\sum_{i=1}^{T}\sum_{j=1}^{k}{\left(d_{ji}-o_{ji}\right)}^{2}$$

where, *T* describes the training samples number, *k* is the output layers number, *d_ji_* and *o_ji_* are the desired value and output value of the CNN respectively. Gradient descent contains the main part of the ordinary BP algorithm; this technique can be trapped easily into the local minimum. This shortcoming can lead to wrong results in some complicated pattern recognition problems [45, 46, 47].

**Figure 3:**
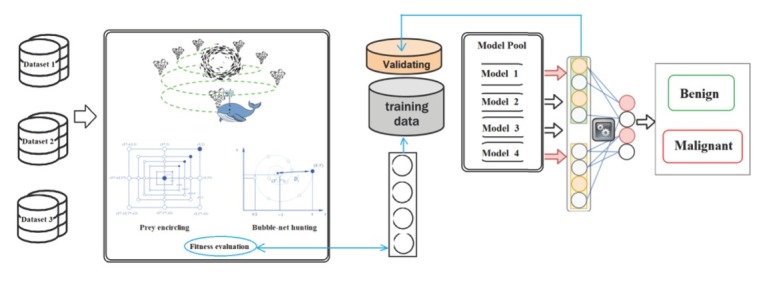
The WOA based framework for the structure of the convolutional neural network

Another advantage of using WOA than the BP for Error minimization is that the WOA based method does not require the backward phase as a high computational cost process. Fig. 3 shows the flowchart diagram of the proposed method.

### 2.3 Dataset Description

Two different dermoscopy databases have been employed for testifying and analyzing the proposed method:

1. DermIS Digital Database [48]: This is an image atlas on different kinds of skin cancers with differential diagnoses which was launched for medical image processing applications. This database is the largest online information service available on the Internet.
2. Dermquest Database [49]: This is an online medical atlas for dermatologists and dermatologist-based healthcare professionals. All images of this database are reviewed and approved by famous international editorial boards. It uses an extensive number of dermatologists and includes over 22,000 clinical images.

**Figure 4:**
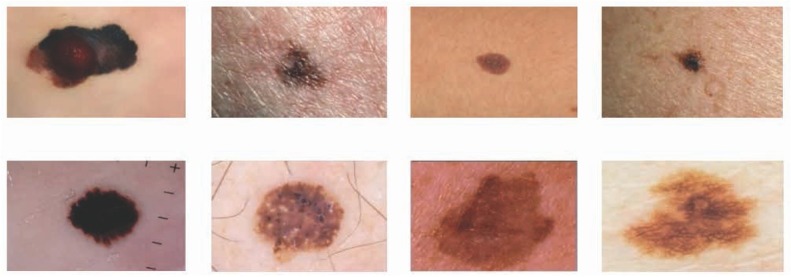
Some examples of the DermIS and the Dermquest databases

Some examples of the databases are shown in Fig. 4.

## 3 Implementation Results

Experimental simulations was implemented using Matlab R2017® software on a Intel Core i7-4790K processor with 32 GB of RAM, and two NVIDIA GeForce GTX Titan X GPU cards with scalable link interface (SLI). The proposed simulations were implemented on two of the standard skin cancer databases to analyze the system performance.

**Figure 5:**
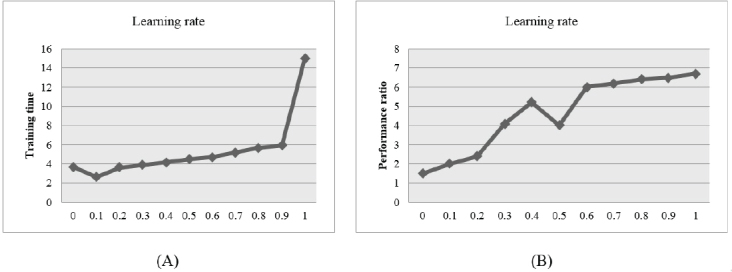
(A) learning rate vs. training time, (B) learning rate vs. performance ratio

70% of data was utilized as the training set and 10% for validating set. The remaining 20% was used as test sets. This division is known as the Pareto principle (80/20 rule) that states that, for many events, roughly 80% of the effects come from 20% of the causes [50]. For determining what images should be included in the training, validating, or testing part, they were selected randomly. For fair image processing, all images of the datasets were resized to 640×480. The proposed CNN was trained by the WOA method. In the presented experiment (Fig. 5), it is obvious that the learning rate varied between 0.2 and 0.9, since the radius and the number of neuron cells are different, almost 100% of training pixels will be included in the prototype neurons.

The best case is to select a neural network with smallest neuron volume. Based on [51], it is possible to select a proper learning rate by the performance ratio. Fig. 5 shows that by increasing the learning rate, both the performance ratio and training time will increase.

However performance ratio is significant, for making a trade-off between performance ratio and training time, the learning rate is selected to be 0.9.

As explained before, Dermis and the Dermquest are employed as two most applicable databases for testifying the proposed method.

30,000 iterations were performed to train the proposed network. For making a correct and independent analysis of the images, the training step was repeated 60 times and the final results were described based on the mean values.

For testifying the performance of the proposed system, five performance metrics were employed and are defined as follows.

(14) $$sensitivity=\frac{Number\;of\;correctly\;detected\;\;skin\;cancer\;\;cases}{Total\;number\;of\;skin\;cancer\;cases}$$

(15) $$specifity\;=\frac{Number\;of\;correctly\;detected\;\;health\;skin\;cases}{total\;number\;of\;healthy\;Skin\;cases\;}\;$$

(16) $$PPV=\frac{Number\;of\;correctly\;\;detected\;skin\;cancer\;cases\;}{Number\;of\;detected\;skin\;cancer\;cases}$$

(17) $$NPV\;=\frac{Number\;of\;correctly\;detected\;healthy\;skin\;cases}{Number\;of\;detected\;healthy\;skin\;cases\;}$$

(18) $$accuracy=\frac{Number\;of\;correctly\;detected\;cases}{total\;number\;of\;cases}$$

There are different research works which have been introduced for skin cancer detection [52, 53, 54]. Each of these methods have their own difficulties and shortcomings. Introducing all of these methods is not possible. Therefore 10 methods have been selected for comparison with our proposed method.

The method of [55] is based on a commercial tool. The method of [56] is about a framework based on the semi-supervised system. For a fair comparison, automatically extracted descriptors of this method are employed. Some deep learning based systems like Ordinary CNN, AlexNet [57], VGG-16[58], ResNet [59] , LIN [60], and Inception-v3 [61] are also utilized for this comparison. Table 1 illustrates a performance comparison between the proposed system and the aforesaid methods.

**Table 1:** Comparison of the performance metrics for skin cancer detection

Method	Performance Metric				
	Sensitivity	Specificity	PPV	NPV	Accuracy
Proposed CNN/WOA Method	0.95	0.92	0.84	0.95	0.91
MED-NODE texture Descriptor[56]	0.64	0.87	0.76	0.79	0.78
MED-NODE color descriptor [56]	0.76	0.74	0.66	0.83	0.75
Spotmole [55]	0.84	0.59	0.58	0.85	0.69
AlexNet [57]	0.84	0.61	0.67	0.85	0.82
ResNet-50 [59]	0.86	0.80	0.71	0.84	0.83
ResNet-101 [59]	0.85	0.77	0.75	0.89	0.85
VGG-16[58]	0.90	0.86	0.79	0.90	0.86
LIN [60]	0.91	0.89	0.80	0.92	0.88
Inception-v3 [61]	0.84	0.65	0.64	0.72	0.84
Ordinary CNN	0.83	0.81	0.77	0.88	0.83

As can be observed, the CNN/WOA method is most accurate when compared with the other 10 aforesaid methods.

This is due to the combination of the CNN with the whale optimization algorithm. Applying this optimization algorithm for the CNN allows it to escape from the local minima. This gives a global minimum for the BP problem in the CNN and addresses better performance for the proposed method.

The results show the effect of using the WOA optimization algorithm on the deep learning framework.

**Figure 6:**
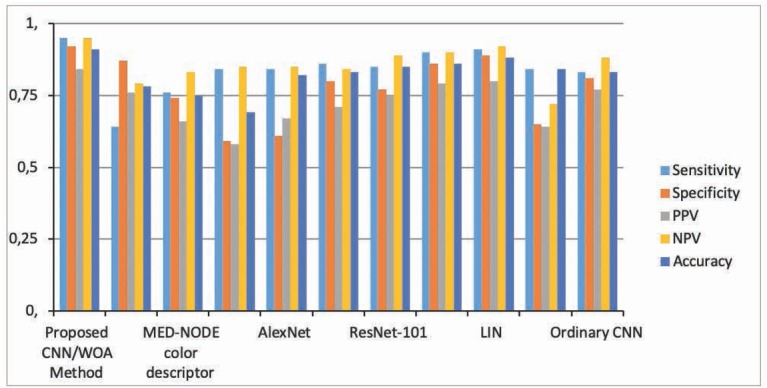
Distribution of classification performance of the methods for skin cancer detection

For more clarification, the distribution of classification performance of the above table is shown in Fig. 6 as a bar chart.

The proposed detection system here has two classes: the background region and the cancerous region. The input layer of the proposed CNN/WOA network contains 3 × n pixel feature vectors which describe the information about R, G, and B of the image. As it is explained before, rectified linear unit (ReLU) is utilized as the activation function of the network. The output layer presents a two labeled image including 0 (background region) or 1 (cancerous region).

**Figure 7:**
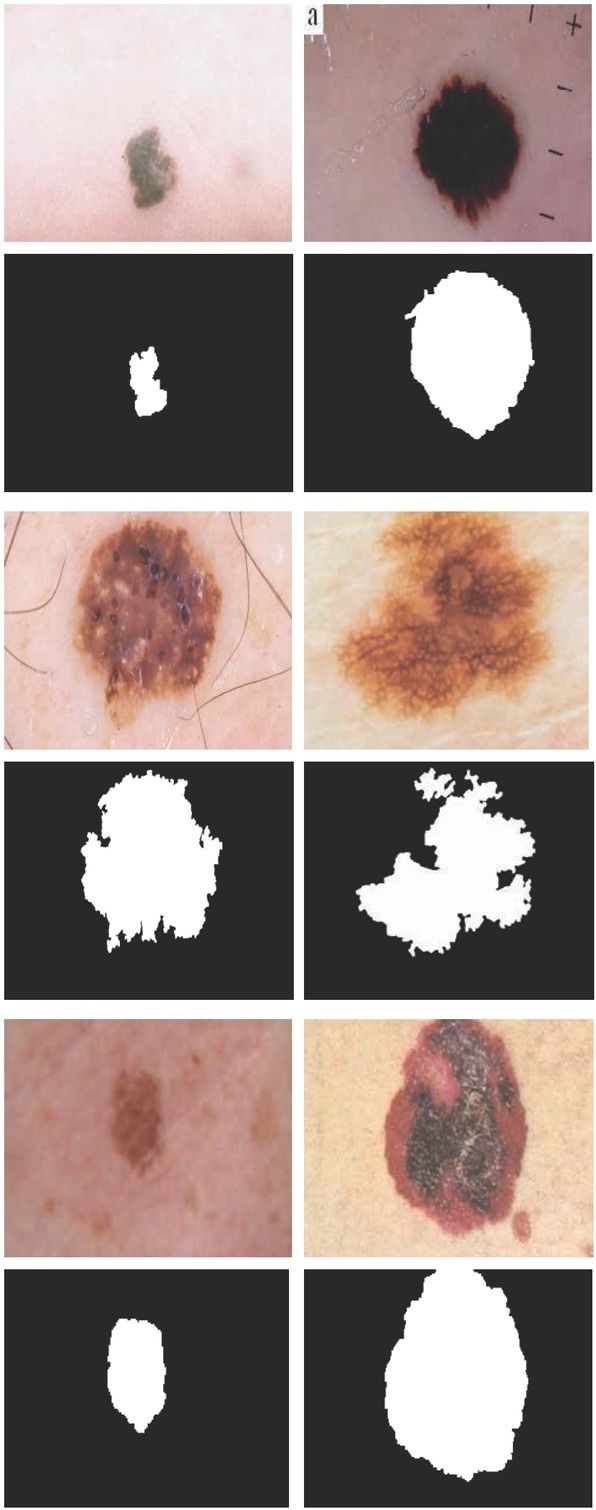
Sample skin cancer detection results: first and third columns: input images, second and fourth column: detected masks based on optimized CNN/WOA method.

Fig. 7 show the results of some samples of the process of the skin cancer detection system detection using the proposed CNN/WOA method. In the figure, the first and the third columns show the original images and the second and the fourth column show the detected masks based on optimized CNN/WOA method. Experimental results show the high efficiency of the presented method for the diagnosis of the skin cancer regions.

## 4 Conclusions

In this paper, a new method is proposed for skin cancer detection. The proposed method uses a meta-heuristic based algorithm for optimization of the Convolutional neural network in training the biases and the weights of the network based on back propagation. To do so, the half-value precision is considered for the proposed optimized CNN as the cost function on a skin cancer validation process which includes a simplified measured error between the reference and the system output. In this study, a recently introduced algorithm called whale optimization algorithm is utilized for minimizing the error rate of the learning step for the Convolutional neural network. The proposed method is called CNN/WOA. The proposed method is then tested on images from two wellknown databases including DermIS Digital Database and Dermquest Database and compared with 10 number of popular classification methods. Final results show the accuracy prominence of the proposed system toward the compared classifiers.
